# Patient perceptions of and experiences with stigma using telehealth for opioid use disorder treatment: a qualitative analysis

**DOI:** 10.1186/s12954-024-01043-5

**Published:** 2024-06-27

**Authors:** Jessica V. Couch, Mackenzie Whitcomb, Bradley M. Buchheit, David A. Dorr, Darren J. Malinoski, P. Todd Korthuis, Sarah S. Ono, Ximena A. Levander

**Affiliations:** 1 School of Medicine, Oregon Health & Science University, Portland, OR USA; 2 Department of Medicine, Division of General Internal Medicine & Geriatrics, Section of Addiction Medicine, Oregon Health & Science University, Portland, OR USA; 3 Department of Family Medicine, Oregon Health & Science University, Portland, OR USA; 4 Department of Medicine, Division of General Internal Medicine & Geriatrics, Oregon Health & Science University, Portland, OR USA; 5 Office of Digital Health, Oregon Health & Science University, Portland, OR USA; 6 Department of Surgery, Oregon Health & Science University, Portland, OR USA; 7 Center to Improve Veteran Involvement in Care (CIVIC), VA Portland Health Care System, Portland, OR USA; 8 Department of Psychiatry, Oregon Health & Science University, Portland, OR USA

**Keywords:** Opioid use disorder, Telehealth, Telemedicine, Buprenorphine, Stigma, Qualitative analysis

## Abstract

**Background:**

Patients with opioid use disorder (OUD) experience various forms of stigma at the individual, public, and structural levels that can affect how they access and engage with healthcare, particularly with medications for OUD treatment. Telehealth is a relatively new form of care delivery for OUD treatment. As reducing stigma surrounding OUD treatment is critical to address ongoing gaps in care, the aim of this study was to explore how telehealth impacts patient experiences of stigma.

**Methods:**

In this qualitative study, we interviewed patients with OUD at a single urban academic medical center consisting of multiple primary care and addiction clinics in Oregon, USA. Participants were eligible if they had (1) at least one virtual visit for OUD between March 2020 and December 2021, and (2) a prescription for buprenorphine not exclusively used for chronic pain. We conducted phone interviews between October and December 2022, then recorded, transcribed, dual-coded, and analyzed using reflexive thematic analysis.

**Results:**

The mean age of participants (*n* = 30) was 40.5 years (range 20–63); 14 were women, 15 were men, and two were transgender, non-binary, or gender-diverse. Participants were 77% white, and 33% had experienced homelessness in the prior six months. We identified four themes regarding how telehealth for OUD treatment shaped patient perceptions of and experiences with stigma at the individual (1), public (2–3), and structural levels (4): (1) Telehealth offers wanted space and improved control over treatment setting; (2) Public stigma and privacy concerns can impact both telehealth and in-person encounters, depending on clinical and personal circumstances; (3) The social distance of telehealth could mitigate or exacerbate perceptions of clinician stigma, depending on both patient and clinician expectations; (4) The increased flexibility of telehealth translated to perceptions of increased clinician trust and respect.

**Conclusions:**

The forms of stigma experienced by individuals with OUD are complex and multifaceted, as are the ways in which those experiences interact with telehealth-based care. The mixed results of this study support policies allowing for a more individualized, patient-centered approach to care delivery that allows patients a choice over how they receive OUD treatment services.

**Supplementary Information:**

The online version contains supplementary material available at 10.1186/s12954-024-01043-5.

## Background

While opioid use disorder (OUD) is understood to be a chronic and treatable condition, the stigmatization of individuals who use drugs and of the medications to treat OUD (buprenorphine and methadone) hinders patients’ access to evidence-based, compassionate care [1–3]. Changes to OUD treatment regulations and policies enacted at the start of the COVID-19 pandemic quickly altered the OUD treatment landscape, allowing for the broad adoption of telehealth (synchronous video and/or audio-only visits) for buprenorphine treatment initiation and continuation [4, 5]. While research is still evolving, these telehealth flexibilities have been associated with increased access, improved treatment retention, and decreased overdose rates [6–8], all of which may serve to narrow long-standing gaps in the OUD cascade of care [9]. Limited data exists to assess how telehealth may impact patient experiences of OUD-related stigma when accessing treatment. Some have theorized that telehealth may mitigate OUD-related stigma by providing a degree of anonymity to patients [4, 10]. However, documented patient perspectives and experiences with stigma when utilizing telehealth for OUD-related care is lacking. Given the rapid and broad implementation of telehealth for OUD treatment, gaining a better understanding of patient perceptions of and experiences with stigma, and the possible impacts on treatment access and engagement in this new healthcare landscape, could help further narrow ongoing gaps in the OUD cascade of care [9].

While diverse theoretical conceptualizations describe social stigma, stigma broadly refers to a multifaceted process in which members of society devalue certain attributes of individuals, and incorporates the interplay of structural disadvantages and socio-cognitive harms that people with these attributes experience [11–13]. In healthcare, stigma is frequently described as occurring at three interacting levels: (1) individual – stigma that is internalized, (2) public – stigma enacted from the community and healthcare professionals, and (3) structural – stigma that underpins discriminatory clinical and institutional policies [3, 14]. On the individual level, internalized stigma contributes to feelings of shame and low self-esteem, reluctance to seek out and remain in treatment, and withholding of drug-related disclosures to avoid sub-standard care [15, 16]. At the public level, stigma can result in adverse social and health-related outcomes for individuals “marked” as having OUD [17]. Public-level stigma can manifest as overt discrimination against and dehumanization of people with OUD, potentially leading to unequal employment opportunities and socioeconomic disparities [1, 18]. Patients openly receiving care in-person at designated OUD treatment centers are especially vulnerable to public stigma [19]. In healthcare settings, stigma may result in lower quality of care and reduce clinicians’ willingness to prescribe medications for OUD [20–22]. On the structural level, the stigma associated with OUD is reinforced by laws criminalizing substance use and highly regulating OUD medications, as well as by clinical policies that deploy punitive care terminations in response to clinic-level program violations, such as missing counselor appointments or having positive urine drug toxicology tests [23–26].

Individual experiences of OUD-related stigma also intersect with institutionalized racism and misogyny. Black and other communities of color have been incarcerated at disproportionately higher rates for drug-related offenses [27], and portrayed less sympathetically when using substances by the mainstream media [28, 29]. Similarly, Black and Hispanic Medicaid and Medicare beneficiaries are less likely to receive buprenorphine for OUD treatment, compared to their white counterparts [30, 31]. Women are also more likely than men to report stigma as a barrier to OUD treatment [32], particularly during the peripartum period [33]. Many U.S. states regard substance use during pregnancy as potential child abuse and grounds for commitment or criminal charges [33]. These patterns contribute to fewer pregnant people utilizing medications for OUD—an especially troubling consequence as overdoses are now a leading cause of death amongst this population [34].

The goal of our study was to explore patients’ perceptions of and experiences with stigma while receiving treatment for OUD using telehealth. While some have framed telehealth for OUD treatment as a possible stigma intervention [10, 35], it is critical to also explore the possibility that telehealth could potentially exacerbate or introduce unexpected stigma experiences.

## Methods

### Study setting and participants

This study was developed as part of a mixed methods sequential explanatory study examining telehealth policy changes, and OUD treatment access and retention in care [36]. Our study took place at a single large urban academic medical center in Portland, Oregon, USA. Participants were recruited from a low-barrier telehealth-only substance use disorder (SUD) clinic, an SUD specialty consult clinic embedded within a primary care clinic, and six primary care clinics that treat patients with OUD. All clinics served the Portland metropolitan area except for the telehealth clinic, which also provided remote care throughout the state. While clinics were within the same center, each developed their own telehealth procedures, including OUD treatment protocols (e.g. frequency of in-person visits, if any; urine drug toxicology testing), at the start of the COVID-19 pandemic. We did not collect data on visits outside our healthcare system, however, we incorporated patient comments on receiving OUD treatment (in-person and telehealth; with methadone and buprenorphine) at other locations into our findings. The Oregon Health & Science University Institutional Review Board approved this study (#23654). We followed the Consolidated criteria for reporting qualitative research (COREQ) (Supplement 1) [37].

We screened potential participants for study eligibility from 831 patients who received OUD care between March 2020 and December 2021. Potential participants were adults between 18 and 89 years old who were identified by their electronic health record (EHR) as having both (1) at least one telehealth visit (video/virtual or phone-only) for OUD with at least one clinic site and (2) a prescription for any formulation of buprenorphine for OUD treatment. Participants were excluded if they endorsed being prescribed buprenorphine exclusively for chronic pain. OUD diagnosis was based on visit ICD-10 codes. Participant information including name, date of birth, medical record number, address, phone number, clinic site, gender, race, and ethnicity were extracted from the EHR into REDCap for the purpose of study recruitment [38]. Clinic leadership from each site authorized the study. Potential participants were contacted about study interest and interviews conducted via phone due to COVID-19 restrictions using number(s) listed in their EHR. We purposively sampled participants across variables of interest, including age, gender, race/ethnicity, clinic site, and rurality [39]. We conducted recruitment, interviews, and analysis in an iterative process as insights were drawn from reviewing interview data.

### Data collection

We collected demographic information both from the EHR and from participant self-report after completing informed consent. When a discrepancy existed, we used self-reported information. Individual quotes are not attributed based on age, gender, or race due to the possibility of identification.

We audio-recorded semi-structured, individual phone interviews conducted at one timepoint after completing informed consent, between October 2022 and February 2023, at a location of participants’ choosing. Audio recordings were professionally transcribed with identifiers removed, then transcriptions were reviewed for quality by at least one research team member. Interviewers were JVC, a female student in medicine and clinical research, and XAL, a female addiction medicine clinician researcher. Both interviewers had prior training in qualitative methods and no prior relationships with participants. Participants were informed of the study purpose and interviewer role at recruitment, during informed consent, and reminded at the start of interviews. No field notes were made. Remaining study team members involved in coding, analysis, and interpretation included MW, a female medical student, SSO, a female qualitative health services researcher and anthropologist, and PTK, a male addiction medicine clinical health services researcher.

Participants received a $40 gift card for interviews, which lasted a mean 40 (range: 13–64) minutes. Interviews included general questions about how patients used telehealth for OUD treatment, how treatment with telehealth compared to in-person, and specific questions about how telehealth affected relationships with their clinical care team and perceptions of stigma. The interview guide and codebook are available in online Supplement 2.

### Analysis

We used an iterative, hybrid inductive-deductive approach guided by reflexive thematic analysis to develop and refine a codebook and code transcripts [40, 41]. After the initial codebook was developed, transcripts were dual coded by two members of the research team (JVC, MW) using Atlas.ti 8 software, with a third team member (XAL) consulting to discuss discrepancies and highlight notable findings. The team met approximately weekly to discuss emerging themes and changes to the codebook and/or interview guide. Quotes assigned to the “stigma”, “privacy” and “patient-clinician relationship” codes were grouped, organized, and analyzed by JVC using the iterative categorization protocol [42]. Thematic saturation was not used as it is not consistent with the reflexive thematic analysis approach [43, 44]. Transcripts and data analysis were not returned to participants for member checking.

## Results

We interviewed 30 study participants (Table 1) with varying substance use histories (Table 2). Mean participant age was 40.5 years (range 20–63). Regarding gender 47% were women, 50% were men and 7% were transgender, non-binary, or gender-diverse; and race/ethnicity (participants could select multiple identities) were 77% white, 13% American-Indian/Alaska Native, 17% Black/African-American, 17% Hispanic/LatinX. Most participants were seen at the low-barrier telehealth SUD clinic (66%), with the remainder receiving buprenorphine through primary care or SUD consult clinics (33%). Housing instability was common, with nine participants (30%) having experienced homelessness in the prior six months.

**Table 1:** Participant demographics

	*N*(= 30)	%
Age (years)		
< 30	7	23%
30–49	15	50%
50+	8	27%
Gender		
Man	15	50%
Woman	14	47%
Other	1	3%
Identified as transgender, non-binary, gender diverse?^a^	2	7%
Race/Ethnicity^b^		
American Indian or Alaskan Native	4	13%
Asian	1	3%
Black or African American	5	17%
Hispanic or Latino/a/x/e	5	17%
Middle Eastern or North African	1	3%
Native Hawaiian or Pacific Islander	0	0%
White	23	77%
Geographic Area		
Portland Metro	10	33%
Other urban (Salem, Eugene)	4	13%
Suburban	11	37%
Rural	5	17%
Education (highest achieved)		
Less than High School	2	7%
High School or GED	8	27%
Some College	14	47%
Associate’s/Bachelor’s	6	20%

^a^ Participants who are men or women could also be transgender, non-binary, or gender diverse

^b^ Participants could select more than one racial/ethnic identity group

**Table 2:** Participant reported substance use histories

	*N*(= 30)	%
**Current non-prescription substance use**		
None	14	47%
Alcohol	9	30%
Cannabis	7	23%
Methamphetamine	1	3%
Opioids	1	3%
**Previous substance(s) of choice** ^a^		
Opioids	30	100%
Alcohol	4	13%
Benzodiazepines	1	3%
Cannabis	1	3%
Cocaine	2	7%
Methamphetamine	4	13%

^a^ Participants could select more than one substance of choice

We organized four themes according to perceptions of and experiences with stigma and telehealth at the (1) individual (theme 1), (2) public (themes 2a, 2b), and (3) structural levels (theme 3).

1. *Individual Level - Telehealth offers wanted space and improved control over treatment setting*.

Many participants described experiencing guilt, shame, and/or anxiety about their OUD, along with physical withdrawal symptoms, that had previously made it difficult, if not impossible, for them to seek care in-person. For these participants, telehealth provided physical and emotional space or distance that could help them overcome anticipated and internalized stigma:

*[E]specially in the beginning, I was very shameful… I felt judged and…demeaned because of my addiction. To be able to speak to someone in the comfort of my own space… and not have to feel…the excessive anxiety that goes with all of that, was absolutely beneficial for me. (1308)*

*I think I was more comfortable going into it being video or online…I didn’t feel like it was as personal at first because you’re not there in-person sweating and talking… I felt like it was easier to get through it, get started with the program in the first place. (1025)*

Telehealth also provided participants with a greater sense of agency, allowing them to seek care from within a known, safe environment, or to quickly end an uncomfortable encounter:

*Telemedicine…it’s not as interpersonal. You’re just a click away from no longer having the interaction and you feel you have more control. You can be in a comfortable environment surrounded by people who make you feel comfortable. (859)*

Some participants noted a preoccupation over whether strangers were judging them during clinic settings—a feeling that, while related to public stigma, is closely tied to and compounded by internalized stigma. By allowing participants to avoid anticipated ‘dread’ and ‘anxiety’ of occupying public spaces, telehealth could reduce experiences of internalized stigma by limiting exposure to these emotional triggers.

*[Telehealth] made [stigma] easier to cope with on a certain extent. ‘Cause I don’t feel I have to air out my problems and be able to go into the office…feeling like shit. Being in the waiting room at every doctor’s office has always been the worst for me…It always feels like people are staring at me…. [with telehealth] I don’t feel as anxious. I don’t feel I have to mentally prepare myself as much ‘cause I’m not socially engaging with all these other strangers. (452)*

### Public level

#### Public stigma and privacy concerns can impact both telehealth and in-person encounters, depending on clinical and personal circumstances

Concerns around privacy and public stigma were described both when participants were engaged with telehealth and with in-person clinical care. Reassurance accessing treatment in-person was heavily influenced by the type of facility where participants received care, notably specialty versus primary care clinics. Participants receiving OUD care in specialty clinics felt that telehealth helped them avoid the public stigma associated with being seen attending an OUD treatment clinic. One participant described, “I was pretty successful…I was connected to pretty important people for what I was doing.…I just didn’t wanna run into anyone in there, that was a stigma to me.” (1304) Some participants described similar concerns around being seen in public, even at primary care clinics; as one noted, “I dread going to their office and being in their waiting room….People are…just nosy….Sometimes you have to run into somebody, and they’re like, ‘What are you here for?’” (376).

Telehealth helped some participants minimize work disruptions by avoiding workplace scrutiny around their OUD treatment. As one participant noted, “[With telehealth, ] I don’t have to miss that much of my day. It’s more private….I can’t really let [my work] know that I slipped up…[with telehealth] I don’t have to ask for time off to go to treatment.” (325).

In contrast, a subset of participants described some privacy concerns relating to using telehealth at-home, due to family, friends, or roommates potentially overhearing. One participant described the clinic as “a more secure environment” and noted, “If I’m in the clinic, then I know that only people that are there to do medical business are there, and aren’t gonna be standing outside the door, listening.” (842) These privacy concerns were particularly pertinent for those who lacked stable housing or who lived in spaces in close proximity to other people. One participant who was living in a college dormitory at the time of their interview noted, “[Maintaining privacy] was kinda hard at college sometimes… because you don’t have a private place you can go to have a doctor’s appointment in college, especially not one on wi-fi….I didn’t want other students to hear me talking about what’s going on.” (408).

#### The social distance of telehealth could mitigate or exacerbate perceptions of clinician stigma, depending on both patient and clinician expectations

Telehealth also mediated participant perceptions of clinician stigma, or discriminatory attitudes or behaviors that their clinicians may harbor. Central to this phenomenon was the feeling of interpersonal and physical disengagement with telehealth, especially with audio-only visits. This disconnection could mitigate or exacerbate interpersonal stigma depending both on participant expectations of how their clinicians would behave if they met in-person, and clinicians’ expectations regarding how involved patients should be in their OUD care.

Some participants expressed the sentiment that, “being there in-person makes it harder for the clinician to just see a diagnosis, a number, and just a face on the computer” (842), and that when, “…you’re in-person and your doctor gets to know you, it’s like there’s an undeniable relationship that happens where they stop seeing you like an addict and start looking at more the whole you.” (1019) One participant, who noted that clinicians seemed, “not very judgmental” in-person, thought that switching to telehealth made them feel more preoccupied around whether their clinician was harboring stigma against them, noting, “You can’t really gauge what [the clinician is] feeling…over the TV screen as much.” (325).

Similarly, other participants noted that certain clinicians’ seemed to behave more suspiciously towards them over telehealth than they would in-person. One participant described their difficulties gaining trust with a clinician whom they had not seen in-person:

*Trying to gain [clinicians’] trust over video chat is a big one. [A clinician] can see [their] patient, but…can’t really ‘get to know them’ get to know them, how they want to…It made it very difficult to gain their trust and respect. But when I went in and met them, it was completely different. Respect each other right off the bat; trust each other. (984)*

Another participant noted how, upon transitioning to telehealth with a clinician whom they had seen regularly prior to the pandemic, their clinician seemed to behave in an increasingly noncollaborative manner:

*[After switching to telehealth, my clinician] kept taking away my medications, not talking to me, and then like not doing anything the same way he’s always done it. I’m frustrated and angry and not feeling heard. When I go into the office, it’s like, ‘Oh, there I am; I’ve showed up.’ It is a feeling like when I only take it on the phone, am I not putting in enough work? (1019)*

In contrast, some participants, particularly those with negative past experiences in the healthcare system, expressed how the distance provided by telehealth could shield them from witnessing discriminatory behaviors enacted by the clinician or other clinic staff. For these participants, telehealth could mitigate perceptions of stigma by making overt expressions of discrimination less visible. As one participant explained, “Maybe it’s because I’m not seeing that person. I’m not looking at them and I’m not like seeing them judging me. Even if they are, it doesn’t come across on the phone….I prefer it.” (562).

A participant who had experienced significant racial discrimination in the healthcare system prior to initiating OUD treatment, discussed how audio-only visits helped overcome hesitation towards interacting with clinicians:

*I haven’t built a lot of trust with any clinician, whether it be over a [virtual] meeting or not…and honestly, this could be a thing that like racially divides. I’ve had really bad experiences with doctors when I was young, so now coming back as an addict, I have some resentment and a lot of hesitation….Body language tells a lot….I think [telehealth] is a little freeing in that…you take that part out of it and you’re just listening to a voice…There’s less hesitancy…because I don’t know what this person looks like or who they are really. (924)*

### Structural Level – The increased flexibility of telehealth translated to perceptions of increased clinician trust and respect

Many participants described feeling burdened by clinic procedural requirements while attending in-person clinics, particularly methadone treatment centers. One recalled, “[Getting methadone] took up so much of my time. They wanted me to take two groups a week, and…go in every day….I was trying to work and I had a new baby. It was really difficult for me.” (996) Another participant, who had attended an outpatient buprenorphine program outside our academic health center with similar requirements to methadone, described, “I found the whole process kind of off-putting and really time consuming. Working full-time made it almost impossible. It was like my life was totally devoted to getting this prescription that was at the time kind of lifesaving.” (859).

Participants who attended such programs often reported a perceived lack of sympathy, if not disdain, for their condition from program staff. Many described being treated like they were, “a bad person being punished,” (991) “a criminal,” (562) or “less than.” (996) One participant who had tried unsuccessfully to get into a withdrawal management center for the first time described, “I’m sitting there to see if a bed opens up for four hours, five hours, and really in a big withdrawal….I just felt like, ‘Oh, this is how they treat drug addicts.’ It’s basically, ‘Okay, we know what you’re going through. Too bad for you.’ (1304).

In contrast, many of the participants who had attended both in-person and telehealth programs perceived that telehealth conveyed a greater level of ‘trust’ and ‘respect.’ As two participants described:

*[My telehealth-based clinic] puts a lot more trust in us as people….I’ve never met them in-person; yet they trust me and I trust them… [when I’ve returned to use], they haven’t treated me like I broke the law, which I felt like this other place did….you feel guilty enough….To have your clinic acting as if they’re angry with you, that’s not helpful. (991)*

*[Telehealth is] so much easier…when you go on Suboxone, it’s like you’re moving up as an adult….You shouldn’t have to go in like a child and do your appointments every five freakin’ days. You should be able to have that mobility [and] flexibility at a certain point….I think that we should have that opportunity to be an adult. (784)*

## Discussion

Stigma surrounding those with OUD and for OUD medications is a widely recognized barrier throughout the addiction cascade of care [14, 45]. While the effects of telehealth on stigma experiences are layered and complex, our findings indicate that telehealth for OUD treatment has the potential to both mitigate and exacerbate patient stigma experiences at the individual, public, and structural levels. Given telehealth’s expanded role for OUD treatment delivery [46] better understanding how telehealth can both negatively and positively impact patient perceptions of and experiences with stigma can help us improve service delivery to potentially further increase OUD treatment initiation and retention.

At the individual level, participants reported that the perceived distance created by telehealth made it easier to overcome feelings of internalized and anticipated stigma to initially access OUD treatment. Higher levels of internalized stigma amongst individuals with OUD may reduce engagement in treatment services [47, 48]. If telehealth mitigates internalized stigma, it could be used as a targeted intervention to increase treatment engagement among individuals for whom stigma is a pervasive barrier, notably women and gender-diverse people [32, 49, 50] as well as people of color [51, 52]. While beyond the scope of this qualitative study, using tools such as the “Brief Opioid Stigma Scale” [53] to examine correlations between internalized stigma and telehealth preference is an important area for future research. Research on other highly stigmatized conditions (e.g. patients living with HIV) suggests that higher perceptions of stigma are associated with an increased likelihood of telehealth use [54]. If telehealth did provide calculable stigma-reduction benefits for individuals with marginalized identities, this could position telehealth as a potential intervention to improve equity in OUD treatment.

On the public level, telehealth impacted participant experiences of stigma from the community and healthcare clinicians, both through its effects on privacy (i.e. the degree of public exposure while seeking treatment) and interpersonal interactions with their clinicians during visits. While most participants found that telehealth provided a greater level of privacy (e.g. from the general public, employers, co-workers, friends, and the like), some participants—particularly those in unstable and/or multi-person housing situations—did not always have access to private spaces for telehealth appointments. While telehealth can allow many receiving OUD care to avoid public scrutiny, the ways in which housing—a key social determinant of health many patients with OUD experience —can affect confidentiality, should be considered in the implementation of telehealth programs for OUD treatment.

Telehealth’s influence on the nature of interpersonal interactions between patients and clinicians also impacted perceptions of stigma in diverse ways. Although telehealth’s potential to create a disconnect between patients and clinicians is often viewed negatively [55, 56], a sentiment shared by some participants in this study, other participants—particularly those who had experienced prior healthcare mistreatment—found that using telehealth helped reduce hypervigilance around monitoring for potential stigma from their clinician. Here, telehealth does not directly address the sources of stigma in the healthcare system, but rather provides a layer of separation. That some participants preferred encounters with physical distance from their clinical team, a finding documented in other telehealth qualitative studies [55], highlights that stigma-reducing “contact” interventions [57], which encourage direct interactions with people with SUD, could have a detrimental impact upon some individuals who the intervention is designed to help. Conversely, some participants found that clinicians seemed more distrustful and stigmatizing over telehealth compared to in-person—a factor that may be shaped by clinician comfort using telehealth. Some clinicians may view in-person meetings as a gesture of hard “work” that demonstrate patients’ commitment to recovery. This erroneous belief may negatively impact how they treat patients receiving telehealth and result in more restrictive prescribing practices [24, 58]. This highlights the critical need for telehealth clinician training and the implementation of telehealth consensus guidelines [59] especially for OUD treatment [60].

On a structural level, many participants perceived increased collaboration and patient-centeredness of telehealth OUD treatment programs, which was tied to the softening of rigid protocols around in-person appointments, meeting attendance, and/or urine drug testing. However, this may be more reflective of specific clinic policies, how these policies were communicated, or how the COVID-19 pandemic overall impacted care, than of telehealth as a care modality. The medical practice of requiring in-person visits and procedures to buprenorphine prescribing can worsen the burden of OUD treatment for patients by compounding patients’ life demands (e.g. attending appointments while balancing work and child care) and straining their resources (e.g. time, transportation costs) [24]. This approach not only perpetuates the structural stigma of OUD by reinforcing disproportionate health burdens when accessing treatment, but can also exacerbate internalized stigma by incorrectly legitimizing views of people with an OUD as less deserving of compassionate treatment. Rigid in-person visit requirements may do more harm than good [61, 62], and our findings lend further support for removing unnecessary treatment requirements as they may be perceived as stigmatizing and reduce interest in initiating or remaining in OUD treatment.

There are important limitations to our study, including that it took place within the context of the COVID-19 pandemic and views on telehealth likely will continue to evolve. Despite using purposive recruitment strategies to include participants from a range of racial/ethnic and geographic backgrounds, the participants were mostly white and urban/suburban dwelling, typical of the study area demographics. This limitation is pertinent given the importance of examining the intersections of race/ethnicity and rurality with OUD-related stigma. Given our reliance on telephone-based recruitment, which was a necessary limitation in light of COVID-19 restrictions, the study sample may under-represent patients with more complex psychosocial issues that would pose a barrier to both engaging in treatment and responding to recruitment [63]. Though it is worth noting, 30% of participants had experienced past six month housing instability. These findings relating to stigma experiences and telehealth emerged from a larger exploratory study examining the impact of telehealth on OUD treatment access. This project was not designed to make comparisons between demographic characteristics or reported levels of perceived stigma. Larger studies with more diverse participants are needed to examine associations between telehealth stigma experiences and particular participant characteristics. Finally, the treatment histories provided by patients spanned a wide range of settings, including but not limited to methadone clinics, inpatient/outpatient OUD specialty clinics, telehealth-based OUD programs, and primary care clinics. It is therefore difficult to make concise generalizations about the impacts of telehealth on patient experiences of stigma when comparing across diverse care settings—this work is worth future research.

## Conclusions

The forms of stigma experienced by individuals with an OUD are complex and multifaceted, as are the ways in which their experiences interact with telehealth. Our results support a more individualized approach to care, whereby patients may choose whether they receive care in-person or via telehealth. Given that aspects of both telehealth and in-person OUD treatment modalities left some participants feeling judged by their clinicians, our findings also highlight the need to further explore how clinicians perpetuate stigma through telehealth-based programs, and how training and clinical guidelines could mediate this. Questions remain regarding how particular patient characteristics (e.g. race/ethnicity, gender, self-stigma) may predict willingness to use telehealth given possible effects on stigma – these are worth exploring in future research. As many participants described telehealth-based programs as perpetuating less stigma due to their more patient-centered clinical policies, in-person clinics should consider adapting similar practices to reduce structural stigma.

### Electronic supplementary material

Below is the link to the electronic supplementary material.

Supplementary Material 1

Supplementary Material 2

Supplementary Material 3

## Data Availability

Due to the often sensitive and private nature of qualitative data and lack of generalizability, raw primary data will not be accessible due to concerns about maintaining privacy and confidentiality.

## References

[1] Dickson-Gomez J, Spector A, Weeks M, Galletly C, McDonald M, Green Montaque HD (2022). You’re not supposed to be on it forever: medications to treat opioid use disorder (MOUD) related stigma among drug treatment providers and people who use opioids. Subst Abuse Res Treat.

[2] Volkow Nora D (2020). Stigma and the toll of addiction. N Engl J Med.

[3] McGinty Emma E, Barry Colleen L (2020). Stigma reduction to combat the addiction crisis — developing an evidence base. N Engl J Med.

[4] Krawczyk N, Rivera BD, King C, Dooling BCE (2023). Pandemic telehealth flexibilities for buprenorphine treatment: a synthesis of evidence and policy implications for expanding opioid use disorder care in the United States. Health Aff Sch.

[5] Krawczyk N, Fingerhood MI, Agus D (2020). Lessons from COVID 19: are we finally ready to make opioid treatment accessible?. J Subst Abuse Treat.

[6] Vakkalanka JP, Lund BC, Ward MM, Arndt S, Field RW, Charlton M (2022). Telehealth utilization is associated with lower risk of discontinuation of buprenorphine: a retrospective cohort study of US veterans. J Gen Intern Med.

[7] Jones CM, Shoff C, Hodges K, Blanco C, Losby JL, Ling SM (2022). Receipt of telehealth services, receipt and retention of medications for opioid use disorder, and medically treated overdose among Medicare beneficiaries before and during the COVID-19 pandemic. JAMA Psychiatry.

[8] Frost MC, Zhang L, Kim HM, Lin L (Allison), editors. Use of and retention on video, telephone, and in-person buprenorphine treatment for opioid use disorder during the COVID-19 pandemic. JAMA Netw Open. 2022;5(10):e2236298. 10.1001/jamanetworkopen.2022.36298 PMC9557869 36223118

[9] Williams AR, Nunes EV, Bisaga A, Levin FR, Olfson M (2019). Development of a cascade of care for responding to the opioid epidemic. Am J Drug Alcohol Abuse.

[10] Molfenter T, Boyle M, Holloway D, Zwick J (2015). Trends in telemedicine use in addiction treatment. Addict Sci Clin Pract.

[11] Goffman E. Stigma: notes on the management of spoiled identity. Prentice-Hall; 1963. p. 147.

[12] Link BG, Phelan JC (2001). Conceptualizing stigma. Annu Rev Sociol.

[13] Moore KE, Wyatt JP, Phillips S, Burke C, Bellamy C, McKee SA (2023). The role of substance use treatment in reducing stigma after release from incarceration: a qualitative analysis. Health Justice.

[14] Cheetham A, Picco L, Barnett A, Lubman DI, Nielsen S (2022). The impact of stigma on people with opioid use disorder, opioid treatment, and policy. Subst Abuse Rehabil.

[15] Woo J, Bhalerao A, Bawor M, Bhatt M, Dennis B, Mouravska N (2017). Don’t judge a book by its cover: a qualitative study of methadone patients’ experiences of stigma. Subst Abuse Res Treat.

[16] Kulesza M, Ramsey S, Brown R, Larimer M (2014). Stigma among individuals with substance use disorders: does it predict substance use, and does it diminish with treatment?. J Addict Behav Ther Rehabil.

[17] Stangl AL, Earnshaw VA, Logie CH (2019). The health stigma and discrimination Framework: a global, crosscutting framework to inform research, intervention development, and policy on health-related stigmas. BMC Med.

[18] Baldwin ML, Marcus SC, De Simone J (2010). Job loss discrimination and former substance use disorders. Drug Alcohol Depend.

[19] Radcliffe P, Stevens A (2008). Are drug treatment services only for thieving junkie scumbags? Drug users and the management of stigmatised identities. Soc Sci Med 1982.

[20] McGinty EE, Stone EM, Kennedy-Hendricks A, Bachhuber MA, Barry CL (2020). Medication for opioid use disorder: a national survey of primary care physicians. Ann Intern Med.

[21] Kennedy-Hendricks A, McGinty EE, Summers A, Krenn S, Fingerhood MI, Barry CL (2022). Effect of exposure to visual campaigns and narrative vignettes on addiction stigma among health care professionals: a randomized clinical trial. JAMA Netw Open.

[22] McLean K, Murphy J, Kruis N (2024). I think we’re getting better but we’re still not there: provider-based stigma and perceived barriers to care for people who use opioids (PWUO). J Subst Use Addict Treat.

[23] Harris J, McElrath K (2012). Methadone as social control: institutionalized stigma and the prospect of recovery. Qual Health Res.

[24] Englander H, Gregg J, Levander XA (2023). Envisioning minimally disruptive opioid use disorder care. J Gen Intern Med.

[25] Doernberg M, Krawczyk N, Agus D, Fingerhood M (2019). Demystifying buprenorphine misuse: has fear of diversion gotten in the way of addressing the opioid crisis?. Subst Abuse.

[26] Simon C, Vincent L, Coulter A, Salazar Z, Voyles N, Roberts L (2022). The methadone manifesto: treatment experiences and policy recommendations from methadone patient activists. Am J Public Health.

[27] Fellner J. United States – punishment and prejudice: racial disparities in the war on drugs. Report No.: Human Rights Watch; 2000 May. p. 183785.

[28] Netherland J, Hansen HB (2016). The war on drugs that wasn’t: wasted whiteness, dirty doctors, and race in media coverage of prescription opioid misuse. Cult Med Psychiatry.

[29] de Benedictis-Kessner J, Hankinson M. How the identity of substance users shapes public opinion on opioid policy. Polit Behav. 2022;1–21. 10.1007/s11109-022-09845-8 PMC9765388 36568520

[30] Barnett ML, Meara E, Lewinson T, Hardy B, Chyn D, Onsando M (2023). Racial inequality in receipt of medications for opioid use disorder. N Engl J Med.

[31] Dunphy CC, Zhang K, Xu L, Guy GP (2022). Racial–ethnic disparities of buprenorphine and vivitrol receipt in Medicaid. Am J Prev Med.

[32] Stringer KL, Baker EH (2018). Stigma as a barrier to substance abuse treatment among those with unmet need: an analysis of parenthood and marital status. J Fam Issues.

[33] Faherty LJ, Stein BD, Terplan M (2020). Consensus guidelines and state policies: the gap between principle and practice at the intersection of substance use and pregnancy. Am J Obstet Gynecol MFM.

[34] Bruzelius E, Martins SS (2022). US trends in drug overdose mortality among pregnant and postpartum persons, 2017–2020. JAMA.

[35] Telehealth a lifeline for patients with substance-use disorders. American Medical Association. Published November 16. 2021. Accessed June 10, 2023. https://www.ama-assn.org/practice-management/digital/telehealth-lifeline-patients-substance-use-disorders.

[36] Levander XA, Foot C, Buchheit BM et al. A retrospective cohort study comparing in-person, video, and phone-based visits for opioid use disorder and loss to treatment. Presented at: AMERSA; November 9, 2022; Boston, MA.

[37] Tong A, Sainsbury P, Craig J (2007). Consolidated criteria for reporting qualitative research (COREQ): a 32-item checklist for interviews and focus groups. Int J Qual Health Care J Int Soc Qual Health Care.

[38] Harris PA, Taylor R, Minor BL, Elliott V, Fernandez M, O’Neal L (2019). The REDCap consortium: building an international community of software platform partners. J Biomed Inf.

[39] Palinkas LA, Mendon SJ, Hamilton AB (2019). Innovations in mixed methods evaluations. Annu Rev Public Health.

[40] Byrne D (2022). A worked example of Braun and Clarke’s approach to reflexive thematic analysis. Qual Quant.

[41] Fereday J, Muir-Cochrane E (2006). Demonstrating rigor using thematic analysis: a hybrid approach of inductive and deductive coding and theme development. Int J Qual Methods.

[42] Neale J (2016). Iterative categorization (IC): a systematic technique for analysing qualitative data. Addict Abingdon Engl.

[43] Corbin J, Strauss A. Basics of qualitative research: techniques and procedures for developing grounded theory, 3rd ed. Thousand Oaks, CA, US: Sage Publications, Inc; 2008. xv, 379 p. (Basics of qualitative research: Techniques and procedures for developing grounded theory, 3rd ed).

[44] Braun V, Clarke V (2021). To saturate or not to saturate? Questioning data saturation as a useful concept for thematic analysis and sample-size rationales. Qual Res Sport Exerc Health.

[45] Socías ME, Volkow N, Wood E (2016). Adopting the cascade of care framework: an opportunity to close the implementation gap in addiction care?. Addict Abingdon Engl.

[46] Centers for Medicare & Medicaid Services Office of Minority Health. Changes in access to medication treatment during COVID-19 telehealth expansion and disparities in telehealth use for Medicare beneficiaries with opioid use disorder [Internet]. Centers for Medicare & Medicaid Services Office of Minority Health. 2022 Jan. Report No.: 28. https://www.cms.gov/files/document/data-highlight-jan-2022.pdf.

[47] Crapanzano KA, Hammarlund R, Ahmad B, Hunsinger N, Kullar R (2018). The association between perceived stigma and substance use disorder treatment outcomes: a review. Subst Abuse Rehabil.

[48] Hammarlund R, Crapanzano K, Luce L, Mulligan L, Ward K (2018). Review of the effects of self-stigma and perceived social stigma on the treatment-seeking decisions of individuals with drug- and alcohol-use disorders. Subst Abuse Rehabil.

[49] Goodyear K, Haass-Koffler CL, Chavanne D (2018). Opioid use and stigma: the role of gender, language and precipitating events. Drug Alcohol Depend.

[50] Perri M, Schmidt RA, Guta A, Kaminski N, Rudzinski K, Strike C (2022). COVID-19 and the opportunity for gender-responsive virtual and remote substance use treatment and harm reduction services. Int J Drug Policy.

[51] Goodyear K, Ahluwalia J, Chavanne D (2022). The impact of race, gender, and heroin use on opioid addiction stigma. J Subst Abuse Treat.

[52] Hollander MAG, Chang CCH, Douaihy AB, Hulsey E, Donohue JM (2021). Racial inequity in medication treatment for opioid use disorder: exploring potential facilitators and barriers to use. Drug Alcohol Depend.

[54] Yang LH, Grivel MM, Anderson B, Bailey GL, Opler M, Wong LY (2019). A new brief opioid stigma scale to assess perceived public attitudes and internalized stigma: evidence for construct validity. J Subst Abuse Treat.

[53] Dandachi D, Dang BN, Lucari B, Teti M, Giordano TP (2020). Exploring the attitude of patients with HIV about using telehealth for HIV care. AIDS Patient Care STDs.

[55] Wyte-Lake T, Cohen DJ, Williams S, Casey D, Chan M, Frank B et al. Patients’ and clinicians’ experiences with in-person, video, and phone modalities for opioid use disorder treatment: a qualitative study. J Gen Intern Med. 2024. 10.1007/s11606-023-08586-6 PMC11347548 38228990

[56] Menage J (2020). Why telemedicine diminishes the doctor-patient relationship. BMJ.

[57] Livingston JD, Milne T, Fang ML, Amari E (2012). The effectiveness of interventions for reducing stigma related to substance use disorders: a systematic review. Addiction.

[58] Bagchi AD, Damas K, Salazar de Noguera N, Melamed B, Menifield C, Baveja A (2022). Comfort with telehealth among residents of an underserved urban area. J Prim Care Community Health.

[59] Lin L (Allison), Fernandez AC, Bonar EE, editors. Telehealth for substance using populations in the age of COVID-19: recommendations to enhance adoption. JAMA Psychiatry. 2020;77(12):1209–10. 10.1001/jamapsychiatry.2020.1698 PMC8108064 32609317

[60] Tele-treatment for substance use disorders | Telehealth.HHS.gov. Accessed May 29. 2024. https://telehealth.hhs.gov/providers/best-practice-guides/telehealth-for-behavioral-health/tele-treatment-for-substance-use-disorders.

[61] Hoffman KA, Foot C, Levander XA (2022). Treatment retention, return to use, and recovery support following COVID-19 relaxation of methadone take-home dosing in two rural opioid treatment programs: a mixed methods analysis. J Subst Abuse Treat.

[62] Ward KM, Scheim A, Wang J, Cocchiaro B, Singley K, Roth AM (2022). Impact of reduced restrictions on buprenorphine prescribing during COVID-19 among patients in a community-based treatment program. Drug Alcohol Depend Rep.

[63] Pertl K, Petluri R, Wiest K (2023). Recruitment challenges for a prospective telehealth cohort study. Contemp Clin Trials Commun.

