# Supplementary material for: Patient perceptions of and experiences with stigma using telehealth for opioid use disorder treatment: a qualitative analysis
Source: Harm Reduct J. 2024 Jun 27;21:125. doi: 10.1186/s12954-024-01043-5 (PMC11210005; doi:10.1186/s12954-024-01043-5)
Supplement: Supplementary file 2 — Supplementary Material 2 [file 12954_2024_1043_MOESM2_ESM.pdf]

**Parent Code**	**Child/grandchild code**	Code ID	Definition	Example Quote
**Levesque - Service level**	**Approachability**		Can people facing healthcare needs actually identify that some form of services exist, can be reached, and have and impact on the health of the individual?: **Examples: outreach, information, transparency, screening**	"I basically started by postin' on my social media asking people I know and the community like, "You guys have any recommendations for medically-assisted recovery options and places that you would recommend?" I didn't really get that many helpful responses through that method. I went on Google and searched. I think I went through <organization> which is an organization that helps connect people to other resources throughout the community. They had a page of addiction-related resources. I called the <short-term telemedicine clinic>. It was one of the first ones that I saw. I did see someone else talk about that clinic specifically in a group that I had been on Facebook. They said that there—that they had a very good experience with the providers there and all of that."(452)
	**Acceptability**		Cultural and social factors determining the possibility for people to accept the aspects of the service (e.g. the social group of providers (race, gender), the beliefs associated to systems of medicine) and the judged appropriateness for the persons to seek care. **Examples: professional values, norms, culture, gender. Include situations where a provider/clinic has specific restrictions around when they will prescribe (i.e. not prescribing to patients who drink alcohol, take benzos, etc)**	"Since at the time, I was takin' benzodiazepines. I cannot find a program that would accept me to get benzodiazepines at the same time. At the clinic I was talkin' to you about before, the place with the doctor and nurse and all that, they do not accept patients who takes benzos as well as opioids 'cause of the risk or something, whatever [unintelligible 04:40]. I was taking benzos at the same time, and I could not find a clinic that would accept both." (243)
	**Availability and accommodation**		Can health services (either physical space or trained providers) be reached both physically and in a timely manner? **Examples: geographic location, accommodation, hours of opening, appointment mechanisms, presence of waivered providers**	"[Telemedicine is] so much easier because I don't have to go anywhere. You know what I mean? I don't have to figure out how this fits in my schedule and what traffic's gonna be like or any of that, so I just tell my girlfriend to stay upstairs, I'm gonna have a meeting and put it on speaker or an appointment. You know what I mean? It's much easier, definitely." (924)
	**Affordability**		The economic capacity for people to spend resources and time to use appropriate resources. It results from direct prices of services and related expenses in addition to opportunity costs related to loss of income. Can vary by type of services and depends on the capacity to generate the resources to pay for care (e.g. mode of payment, mobilization of resources). **Examples: Direct costs, indirect costs, opportunity costs, insurance/payment mechanisms**	Interviewer: "What, if anything, would you like to see change in the future?" Interviewee:"More equitable access, 'cause I know a lot of people that would want to be on Suboxone and do medically-assisted recovery. Except they don't have a phone or insurance."(452)

	**Appropriateness**	"Fit" between services and clients' needs, its timeliness, the amount of care spent in addressing health problems and determining the correct treatment, and the technical and interpersonal quality of the services provided: **Examples: technical and interpersonal quality, adequacy, coordination and continuity. Will include clinic protocols, consistency or inconsistency in providers, perceived comprehensiveness**	"I wish I would feel closer to [the providers]. I don't feel as close because it is virtual. I'm a very hands-on person. I feel better when I'm communicating with people face-to-face... As far as people-to-people, I would love to sit down in person—and face-to-face with one person and connect with them that way, like face-to-face, or F2F, as they say. I like that connection better. When telemedicine started up, I knew I wasn't gonna have a bond with my doctors because not only were they changing them so much, but they were not in my face. I knew it was hard for me to get that connection goin' like I had with my doctor when I was at <Clinic> in person. Then they don't have counselin' available, so I didn't have a counselor to talk to. Now I have counselin, but they're not related to the clinic. It's another whole place, a whole sub-buildin', a whole 'nother company, and I see him face-to-face, like that. That's one of the downfalls is the lack of closeness 'cause of the lack of face-to-face communication. (243)
**Levesque - Patient ability level**	**Ability to perceive**	Ability to perceive need for care, as determined by factors such as health literacy, knowledge about health services, and beliefs related to health and sickness. **Examples: Health literacy, health beliefs, trust and expectations --> This applies specifically to the moment when a patient is initially identifying that they needed to get care for OUD**	"Honestly, up until Covid. I didn't have any experiences with telemedicine. That might have been because, during the course of my addiction, which lasted roughly six years, I didn't go to the doctor a lot. I didn't do check-ups, that kind of thing. The stuff you should to stay healthy, or make sure you're not dying. I may have just not been paying attention to what I had access to. But up until the start of Covid was really when I started using telemedicine or realized that I had access to it. It pretty much changed the way that I go to the doctor entirely." (859)
	**Ability to seek**	Relates to concepts of personal autonomy and capacity to choose to seek care, knowledge about health care options and individual rights that would determine expressing the intention to obtain healthcare. A good example would be female discrimination regarding the initiation of care, or **abuse and neglect discouraging ethnic minorities to seek care. This relates to the challenge of ensuring that care meets the needs of different cultural, socioeconomically disadvantaged, and vulnerable populations**. **Examples: Personal and social values, culture, gender, autonomy**	"I've had really bad experiences with doctors when I was young, so now coming back as an addict, I have a lot of—some resentment and a lot of hesitation going forward with doctors, so I don't really know if I trust any of them just because of the way I've been treated in the past. Like even before I had a problem, like as a child, it just wasn't a good experience for me [laughter]." (924)
	**Ability to reach**	Relates to notion of personal mobility and availability of transportation, occupational flexibility, and knowledge about health care services that would enable one person to physicially reach said providers. **Examples: living environments, personal mobility, transportation, occupational flexibility, social support, knowledge of technology that enable a person to connect via telemed, access to phone/other devices**	"[Telemedicine] was definitely incredibly helpful and has been because I have really bad chronic depression and anxiety. I also have epilepsy. Just all of that combined, can make it hard for me to get to the actual doctor's office, especially on time. I don't have access to a vehicle. I have to take transit every where, which also costs money that I just don't have." (452)

	**Ability to pay**	The capacity to generate economic resources - through income, savings, borrowing or loans - to pa for health care services without catastrophic expenditure of resources required for basic necessities: **Examples: income, assets, social capital, health insurance**	"I know a lot of people that would want to be on Suboxone and do medically-assisted recovery. Except they don't have a phone or insurance." (452)
	**Ability to engage**	Participation and involvement of the client in decision-making and treatment decisions, which is in turn strongly determined by capacity and motivation to participate in care and commit to its completion. This dimension is strongly related to the capacity to communicate as well as notions of health literacy, self-efficacy, adn self-management in addition to the importance of receiving care that is actually appropriate for the person, given its resources and skills. **Examples: Empowerment, information, adherence, caregiver support**	"I think and at the time, like I said, I was working full time, getting through most of my addiction. It was really, really difficult to me to go through the programs that the outpatient clinics in my area offered. Telemedicine was—I was really looking for something where I could talk to somebody once a month, and I had a care advocate, and I could message somebody if I was feeling some type of way. But otherwise, I could just get a script for the medicine and use it the way that I needed to use it—that I was supposed to use it—and go on with my life. That's what I did. And it worked out really well." (859)
**Modifying components to patient access**	**Patient-provider relationship**	Nature and quality of patient's relationship with **provider and/or clinic**. **Related to notions of trust and interpersonal interactions.** ***Should be dual coded with Levesque framework***	"I think it's been different with different practitioners. I think getting back to. I feel I have more trust in <Long term telemedicine SUD clinic> than I do any given one of my practitioners. I trust that they've hired people that are there to try and help people like me. I've had a couple practitioners, really just one in particular, that I didn't actually feel that way about. But, I wasn't too concerned about the repercussions about that. Our appointments were very short. And it was, once again, I was there to get my script and move on." (859)
	**Community/peer support**	Peer = persons with lived experience of SUD. Community/peer support relates to the ability to have positive social interactions with other peers (e.g. recovery groups, group therapy, etc) in/around the clinic. Quotes relating to relationships with clinic staff should go under patient-provider relationship	"I think when I went to outpatient it felt a lot more like, They set up this whole program. I had all of these people that I was meeting with. It was very consistent. Like, this is my counselor. This is my doctor. This is my sponsor. It gave me this whole group of people. Telemedicine has so far not provided that at all. It's more just been we have the ability to prescribe you this drug, as long as you participate in our organization. You come to our organization, then we can prescribe it to you. You can figure out how to do that on your own." (859)
	**Stigma**	Should include responses to the interview question about whether a patient endorses experiencing stigma and if/how telemedicine affected that, as well as patients' other reported experiences of stigma in healthcare. **Consider avoiding dual-coding with Levesque in order to avoid overlapping quotations betwen stigma and access papers?**	"I haven't built a lot of trust with any provider, whether it be over a zoom meeting or not [unintelligible 07:11], so—and honestly, this could be a thing that like racially divides. I've had really bad experiences with doctors saying horrible things to me. Before I became an addict and I was a child, I had doctors accusing me of stealing needles, a bunch of weird stuff, like I tore my MCL twice and never got any sort of pain medication because they felt that if I wanted pain medication for my torn knee, that that made me a drug seeker. You know what I mean?" (924)

	**Technology**	Any mention of technology use including issues with technology, experiences with technology, attitudes toward technology, etc. **Should be dual-coded with Levesque framework**	Interviewer: "Have any technical issues come up during your appointments?" Interviewee: "Well, yeah, definitely. Actually I remember specifically when I was with <School> there was audio issues. The image was very blurry. Then there was a lot audio issues that had to be corrected throughout the appointment. But nothing that prevented the appointment from happening." (859)
**Additional codes related to interview questions**	**Previous OUD experience**	**NOT telemedicine related.** Includes prior treatment and context of their OUD course and history; **do not need to do Levesque access coding for anything prior to COVID-19 related changes**	"I originally tried to go through an outpatient program. They required multiple meetings a week. They required counseling at the program, multiple NA-related meetings a week, and weekly checkups with my doctor who would only fill my prescription for Suboxone once a week. I had to make sure I took all of those steps in order to do that. There was regular drug testing, not that that was an issue 'cause I was pretty determined to quit at the time." (859)
	**New to OUD treatment with telemedicine**	Someone who does not have prior OUD treatment experience pre-COVID/pre-telemedicine i.e. their first experience with OUD treatment was with Tele-OUD, thus they can't compare TeleOUD to other past experiences.	Interviewer:"What was the first setting or the first clinic that you went to to get treatment?" Interviewee: "The <short-term telemedicine clinic>, I think." Interviewer: "Have you been anywhere that did inpatient or in-person treatment? " Interviewee: "No." Interviewer: "Have you been anywhere other than <short-term telemedicine clinic>?" Interviewee: "No." Interviewer: "<Short-term telemed clinic> was the first time that you experienced getting suboxone or buprenorphine prescribed for you?" Interviewee: "I think so, yes." (370)
	**Disruptions to treatment**	Participant mentions any issues that resulted in a disruption of their care, whether they were on the patient's side or the clinic's (e.g. insurance, doctor changed/moved, psychosocial stressors, return to use, etc)	Interviewer: "What disruptions, if any, have you had in getting your buprenorphine since you first started it?" Interviewee: "Any disruption I've had has just—there has been sometimes where there's an error made on the MyChart for the prescription refill, or the provider sent it to the wrong pharmacy. It's been kind of a pain in the ass before to have that prescription sent back to the right one or trying to figure out what pharmacy it went to in the first place. Also, just not being told in advance if there's gonna be an office closure or something." (452)
	**Privacy**	Includes responses to question about how a patient maintains their privacy during telemedicine visits, AND any other general references that a patient makes to concerns around privacy in healthcare	Interviewer: "...how do you maintain privacy during telemedicine visits, whether it's for group therapy or—" Interviewee: "We have two bedrooms, so I go to the other room. You know what I mean? If it's just a meeting with me and a counselor, I don't care if my girlfriend's around because she knows everything, but if I'm in a [group recovery] meeting, I'm going to a place where she can't be. You know what I mean? She knows that, and she knows how I feel about it, so I don't want to cross that boundary with anyone else because it's something that, you know, "do unto others", I do not appreciate that." (924)

**Behavioral health**	Includes responses to interview question about how a patient received BH services during COVID, as well as an other references made to how a person did or did not receive BH support and their experiences with it	"Covid made it super hard to find counselors via telemedicine who weren't completely booked. That wasn't something that was provided by my health provider. Addiction recovery counseling I think was—I had access to it to some degree. But behavioral counseling was very hard to access." (859)
**Lab-work**	Any references to how a patient has received labwork (bloodwork, UDS, etc) pre- and post-COVID	Interviewer: I wanted to ask how have you gotten any lab work you need to get done such as urine drug tests or bloodwork?" Interviewee: I've gone to my primary care clinic <primary care clinic> 'Cause I'd also get my Suboxone through there as well. I have gone over there when I have needed to get my bloodwork done. 'Cause it's pretty convenient for me to get to, for the most part." (452)
**Pharmacy**	Any references to how a patient has interacted with a pharmacy	Interviewer: "I was gonna ask you how has it gone picking up your buprenorphine prescriptions at a pharmacy, after you've done a telemedicine visit for opioid-use disorder?" Interviewee: It usually goes fine. That instance of that chick, it's really only been with that specific person. Also, people, other people, waiting in line for their prescriptions overhearing what I'm getting. Right after getting my appointment done, it usually doesn't take very long at all to get the prescription sent over to the pharmacy. They're open pretty late on weekdays. I haven't really faced any difficulty getting it 'cause I also have had it delivered before 'cause they have the delivery option." (452)
**Sexual health/infectious disease counseling**	Patients responding to prompts around comfort with sexual health/sexual disease counseling via telemedicine	Interviewer: "What would you think, theoretically, about getting sexual health counseling or having discussions around infectious diseases via telemedicine?" Interviewee: "I think that that would be really helpful. For the same reasons that I think that I felt less—it was a lot easier to call someone or talk to them on a computer about my addiction issues—it might be easier to talk about sexual issues or sexual health issues." (859)
**Narcan/naloxone**	Patients responding to prompts around how they received Narcan/naloxone during the pandemic	Interviewer: "Thinking about naloxone or Narcan. Have you gotten that during the pandemic?" Interviewee: It's been offered to me. I haven't picked up any because I haven't used or been using. I did have a practitioner offer to prescribe it to me so that I could pick it up at a pharmacy." (859)
**In-person vs telemedicine balance**	Includes responses to interview question about what percentage of their visits they would prefer to be in-person versus telemedicine, and other references to **specific situations where telemedicine or in-person care would be preferred/ necessary**	“There hasn’t really been any downfalls from switching from telemedicine to in-person. The only thing I can think of, was my doctor wasn’t going to prescribe it because I just became her patient and she wanted to see me in-person. I couldn’t do it for at least two weeks, so we came to an agreement. I would go in and see her, so I could get the medicine and meet in-person. That’s pretty much all I can think of with that one.” (984)

	**Additional services desired**	Includes responses to the interview question, "What other health care services would you like to receive along with OUD treatment via telemedicine?" As wass as any instance where patient refers to additional services that they would like to receive via telemedicine	Interviewer: "What other health care services would you like to receive along with opioid use disorder treatment via telemedicine?" Interviewee: "...I see my GP regularly through telemedicine. Behavioral counseling I think would be super beneficial. Of course, that does exist. It's just something that I need to get back on track with....Honestly, I think even dental would be cool. Because every now and then I have a pain in my tooth. I'm like, "Do I need to go in for this?" Just quick 15 minute pre-screening type thing would be cool." (859)
**Other codes**	**Overall satisfaction**	A participant makes some reference to their overall satisfaction with Tele-OUD	"In-person and via a video call were actually successful; both were good. I haven't really had a negative, unsuccessful video chat or in-person. Just trying to get a doctor, that has been unsuccessful. But seeing a doctor for that both video chat and in-person, both good honestly." (984)
	**Statment of gratitude**	A participant makes some statement expressing gratitude for the services that they have received	"I definitely would say that telemedicine saved my life. Because when I started Suboxone, I was homeless and just really depressed and couldn't go—you couldn't go anywhere 'cause of the lockdown....I definitely am happy that it's a thing 'cause of all of the reasons I listed." (452)
	**Notable quote**	Used to flag quotes that we would especially like to include in the paper	
