# Supplementary material for: Patient perceptions of and experiences with stigma using telehealth for opioid use disorder treatment: a qualitative analysis
Source: Harm Reduct J. 2024 Jun 27;21:125. doi: 10.1186/s12954-024-01043-5 (PMC11210005; doi:10.1186/s12954-024-01043-5)
Supplement: Supplementary file 3 — Supplementary Material 3 [file 12954_2024_1043_MOESM3_ESM.pdf]

Semi-structured Interview Guide - Patient Participants
***Project Title**:* Telemedicine for Treatment of Opioid Use Disorder (Tele-OUD) in the OHSU Health System

Introduction: Hello, my name is [insert name] and I am the [research assistant/study physician]. You have a valuable perspective that can be used to help us better understand the use of telemedicine for treating those with opioid use disorder. Telemedicine is when you get health care by physicians or other health care practitioners from a distance using video and/or audio.

This interview is confidential, answers will not be shared with your medical team, and should take between 45-60 minutes. Prior to asking you any questions I would like to go over the informed consent. **[Go to Informed Consent/Information Sheet].** Thank you.

I'd first like to start by asking a few brief questions about you. **[Go to Demographic Survey]**.

Doing this interview is your choice. You can stop or take a pause if you need to do so. During the interview, I'm going to ask you some questions. You can take as much time as you need to answer. You can skip a question if you want to come back to it later, or skip it completely if you do not wish to answer. You don't need to share anything you don't want to share. There is no right or wrong answer to any question.

We want to learn about the experiences you have had with telemedicine for opioid use disorder. We want to hear your stories and colorful answers are more helpful than short answers. Give us as much detail as you can so you can "take us there," as though we were watching a movie. Your stories and opinions will help us better understand telemedicine to shape future patient care.

What questions do you have before we begin?
Are you ready to start the interview?
For the purposes of our study "Telemedicine" is when you get health care by physicians or other health care practitioners from a distance using video also known as virtual and/or audio visits done by telephone.

This part of the interview is being audio-recorded for research study purposes only so I can focus on what you are saying and to make sure we do not miss any of the important things that you say. I am now turning on the audio recorder. **[Turn on Recorder]**
This is participant ______ [participant number] on [date] and [time].

<u>INTERVIEW QUESTIONS</u>
<u>TELEMEDICINE EXPERIENCES</u>
I want to hear your story of how telemedicine has affected your opioid use disorder treatment. Please walk me through your experiences with telemedicine for treatment of opioid use disorder. If you've had many different experiences with telemedicine, you can focus

on the most memorable one, however we appreciate all the experiences you want to share. *<Additional probes below as needed>*

- Tell me about a time that telemedicine did not work well for you? How did that go? What do you think could have made that experience work better for you?
- Tell me about a time that telemedicine worked well for you? How did that go? What do you think made that experience work well for you?
- Things have changed a lot over the past couple years when it comes to telemedicine, tell me about how your experiences with telemedicine have changed over time. *<ask for experiences earlier or later in the pandemic depending on earlier responses>*
- What disruptions, if any, have you had in getting your buprenorphine (suboxone, Subutex, sublocade)? What role did telemedicine play in that disruption? What do you think could have prevented that from happening?
- What's been harder in your life when using telemedicine?
- What're the negatives or downsides to telemedicine? What do you miss? What do you not like about telemedicine?
- What's been easier in your life when using telemedicine?
- What're the positives or upsides to telemedicine? What do you want to keep the same? What do you like about telemedicine?
- How do you maintain your privacy for your telemedicine visits?
- *<If minimal pre-telemedicine experiences then>* What was difficult or challenging about accessing healthcare or going to the doctor before you started using telemedicine?
- What were your experiences with treatment for opioid use disorder before telemedicine? How long have you been in treatment? What types of prior treatment experiences have you had?

Tell me about some of your experiences with treatment for opioid use disorder before using telemedicine. If you've had many different experiences, you can focus on the most memorable one, however we appreciate all the experiences you want to share. Go back to above prompts to ask about similar experiences with in-person.

<If not already covered fully above> **Tell me about how telemedicine has affected your relationship with your provider and your clinic** . *<Additional probes below as needed>*

- How does treatment with telemedicine differ from when you were going in-person to the clinic to get treatment? What about difference between phone (audio only) and virtual (video)?
- How do you think your relationship with your providers/healthcare team has changed because of telemedicine? *<If prior in-person experience>* How did knowing your provider before you started doing telemedicine impact your ability to maintain a connection?
- Given the distance between you and your provider when using telemedicine, how do you build and maintain trust with them?
- A lot of people with opioid use disorder mention concerns around feelings of stigma in the health care system – Is that something that resonates with you? How has

telemedicine potentially changed your experiences with stigma in the healthcare system?

<If not already covered fully above> **Tell me about your experiences with using the technology (phone, tablet, computer, etc.) you need during your telemedicine visits.** *<Additional probes below as needed, if no use of telemedicine can jump to question about "what technology would you need…">*

- Have you used MyChart (the online patient portal/online program)?
  - *<if yes>* Walk me through how use MyChart? What do you use it for? What issues, if any, have you had with using MyChart? How could that program be made better?
  - *<if no>* What has kept you from being able to use MyChart?
- How did you get the technology you needed set-up for your first telemedicine visit?
- How do you troubleshoot any technology issues that come up during your visits?
- What technology would you need to make telemedicine visits work (better) for you?
- What support or assistance would make telemedicine visits work (better) for you?
- What has prevented you from having a telemedicine visit with your provider?
- When you look back to what your clinic was asking you to do to connect with your provider via telemedicine, what technology did they expect you to have available? What did they expect you to know how to do with that technology to connect with your provider? Did you feel that these expectations were reasonable?

Tell me about how you have gotten <u>other</u> services related to opioid use disorder treatment since the start of the pandemic for example counseling, lab work, pharmacy services. *<Additional probes below as needed>*

- How have you been getting behavioral health/counseling (in-person, video, and/or audio)? How have those visits gone for you?
- How have you gotten any lab work you need done such as urine drug tests or blood work during the pandemic? How has that process changed since before the pandemic?
- How have you been getting naloxone (Narcan) during the pandemic?
- How has it gone picking up your buprenorphine prescriptions at a pharmacy after a telemedicine visit for opioid use disorder? How has that process changed?

Telemedicine services are relatively new, what other health care services would you like to receive along with opioid use disorder treatment via telemedicine? *<Additional probes below as needed>*

- What would you think about getting sexual health counseling and discussions around infectious diseases such as HIV, hep C, syphilis, via telemedicine?
- *<For those who can become pregnant>* What would you think about getting reproductive health and family planning counseling via telemedicine? What about counseling on contraception and possibly prescriptions for the pill, patch, or ring?

<u>REFLECTION AND WRAP-UP</u>

Thank you so much for sharing your experiences. I have a few last questions asking you to reflect on your overall experiences. Then we're done.

- **Looking back over the past couple of years, do you wish anything had played out differently in terms of treatment of your opioid use disorder? What changes would you make if you could go back and redo things?**

- **In an ideal world, of all the visits you have with your doctors for your opioid use disorder treatment, what percent would you want to be in-person versus using telemedicine (phone and/or video)? Why is that the right amount/division of in-person versus telemedicine for you?**

- **The ability to prescribe buprenorphine using telemedicine is new since the start of the pandemic – What would you like to see continue? What would you like to see change in the future?**

- **Thinking back on all you've told me today – what is the most important message you want the research team to know from your story?**

Thank you, again, so much for your willingness to share your stories with me. **Is there anything else you would like to add? Are there any questions you have for me? End of Interview [Turn off Recorder]** I have turned off the recorder.

Contact information for how to reach the study team is included in the information sheet we reviewed earlier. If you think of anything else that you would like to add please contact us. Here is your $40 gift card for your participation in this study.
